# Dealings between Cataract and Retinal Reattachment Surgery in PVR

**DOI:** 10.1155/2016/2384312

**Published:** 2016-02-29

**Authors:** Svenja Deuchler, Pankaj Singh, Michael Müller, Thomas Kohnen, Hanns Ackermann, Joerg Iwanczuk, Rachid Benjilali, Frank Koch

**Affiliations:** ^1^Vitreoretinal Unit, University Eye Hospital, Frankfurt/Main, 60590 Hessen, Germany; ^2^University Eye Hospital, Frankfurt/Main, 60590 Hessen, Germany; ^3^Institute of Biostatistics and Mathematical Modelling, University Hospital, Frankfurt/Main, 60590 Hessen, Germany; ^4^Oculus Optikgeräte GmbH, Wetzlar, 35582 Hessen, Germany

## Abstract

*Introduction*. To evaluate the impact of the eye lens status and oil side effects on the outcome of vitreoretinal surgery in retinal detachment with proliferative vitreoretinopathy (PVR) and a temporary silicone oil tamponade (SOT).* Methods*. 101 eyes were analyzed retrospectively and 103 eyes prospectively in regard to their retinal reattachment success rate and key factors for the outcome. Subgroup analysis of 27 eyes with Scheimpflug lens photography (SLP) before and after retinal reattachment service with SOT was performed. For SLP (65% phakic eyes) a Pentacam densitometry reference body with 3 mm diameter was chosen and 3 segments (anterior/mid/posterior) were evaluated separately after a quality check.* Results*. The retinal reattachment rate was highest in the prospective pseudophakic group (*p* = 0.039). Lens transparency loss occurred earlier in middle aged patients than in younger patients. Besides the nucleus, layers posterior and anterior to it showed specific transparency changes. The emulsification rate was higher when eyes had been operated on in the anterior chamber before retinal reattachment service.* Conclusions*. Retinal reattachment surgery seems to benefit from preoperative cataract removal. We found significant lens changes in the nucleus as well as in the layers anterior and posterior to it. This corresponds to the histology of the lens epithelium published before.

## 1. Introduction

In retinal detachment surgery considerations for the optimal surgical procedure include vitreous substitutes as well as lens aspects: gases or silicone oils are important tools in vitreoretinal surgery because they have the ability to displace aqueous humor from the retinal surface while maintaining the adhesion between retina and retinal pigment epithelium. At the same time, the increase of oxygen concentration after removal of a significant portion of vitreous accelerates lens transparency losses. Several questions are raised upon this complex chapter in ophthalmology: Should younger patients be operated on externally, for example, using buckling technology to seal a retinal hole [1–4]? Is the pneumatic retinopexy procedure justified in spite of a lower primary success rate, because there is a significantly lower rate of lens transparency loss [5]? What kind of impact does pars plana vitrectomy have on the aging process of the patients lens and what is the best choice between different gases and silicone oils [6–9]?

Do lenses primarily have to be removed once there is pathology associated with a retinal detachment, for example, an anterior hyaloid fibrovascular proliferation process, which handicaps the surgeon in removing membranes in the outermost periphery of the vitreous cavity?

It is probably too much simplified but still justified to summarize the current attitude of many specialists in the community: they prefer to save the lens in younger patients, especially where a clearly defined single hole or a group of holes easily accessible with an external buckle could encourage us to stay outside the vitreous or choose a pneumatic retinopexy strategy to seal single breaks in the upper part of the eye. However, when condensed vitreous is obviously pulling up the retina in many places especially when mixed up with blood and pigment cells and when holes cannot be clearly defined or excluded, this should lead us to immediate intraocular intervention with careful pars plana vitrectomy as complete as possible often combined with or following cataract surgery.

The complexity of the pathology will drive our decision on how to substitute the vitreous with either a special physiologic formula of water (e.g., balanced salt solution), air, several expanding gases, or even several silicone oils.

Because oils can be used as a temporary, prolonged, or even permanent tamponade, it seems to be suitable especially for the management of complicated retinal detachment due to PVR or viral retinitis, giant retinal tears, trauma, and severe proliferative diabetic retinopathy [10, 11], retinal detachment due to a macular hole in highly myopic eye [12, 13], chronic and persistent macular holes, colobomatous retinal detachment [14], and chronic uveitis with hypotony [15]. In all these cases we are still seeking for more knowledge about the best timing for cataract surgery since lenses might be a helpful diaphragm between posterior and anterior chambers, an obstacle for adequate observation, or “just in the way,” hindering the surgeon to complete the PPV if needed.

## 2. Material and Methods

### 2.1. Study Population

In an extensive retrospective and prospective study set-up (*n* = 204 completed data sets) we analyzed eyes suffering from a retinal detachment with P(D)VR in regard to their retinal reattachment success rate and key factors for the outcome. The main goal was to lower the retinal redetachment rate. Other goals included gathering information about the lens status and its role in the outcome of retinal reattachment service and analyzing whether and how silicone oil emulsification over a 4-month fill according to the algorithm [16–18] of our standard operation procedure (SOP) affected the outcome.

The study was approved by the ethical review committee of the University of Frankfurt/M (IRB decision number E 190/11, transaction number 403/11). The ethical review committee also approved the written participant consent, which was provided by all participants.

This study was conducted in accordance with the Tenets of the Declaration of Helsinki. Patients' records were pseudonymized and deidentified prior to statistical analysis.

### 2.2. Changes in Lens Density

Retrospectively, 28.71% of the patients were pseudophakic from the beginning, cataract surgery was provided simultaneously with the oil fill in 7.92% or with the oil removal in 34.65%, and in 23.76% of eyes cataract surgery was provided later on.

Prospectively, 39.81% of pseudophakic patients were included, 2.91% of the patients were operated on simultaneously with the oil fill and 39.81% with the oil removal. 14.56% of the patients stayed phakic. The primary phakic patients received Scheimpflug lens photography (SLP) before and after retinal reattachment service with SOT.

To measure changes of lens densities (densitometry) prospectively we used the Pentacam HR Scheimpflug anterior segment imaging system (Oculus Optikgeräte GmbH) Version 1.20b67 [19–21]. It is a noninvasive device; no contact with the eye is necessary. When we measured lenses with a Scheimpflug camera, our measurements followed a geometric principle based on the “non-parallel-to-each-other” orientation of lens and image planes. In the consequence, targets in different distances to a camera will all be captured in focus and distortion-free which plays an important role in ophthalmology for selective measurement especially on the cornea and on the lens. The eyes had to be dilated at least 5 mm to stay in the study group. Primarily, we had 62 phakic patients, who were included in the prospective part of the retinal detachment study. Before the statistical analysis we checked the data for plausibility: quality and completeness of data sets (pre- and postoperative examinations). For quality check only pictures that met the quality specification of the Pentacam imaging system (e.g., no blinking or hazy cornea) were included for analysis. After the quality checks, 27 eyes stayed in the final analysis. The reference body was centered to the corneal apex with a diameter of 3 mm to analyze the lens densitometry average value with a 3D model for the whole lens as well as for the 3 lens segments (anterior/mid/posterior) separately as explained below.

To detect potentially age-related lens changes, we grouped the eyes into group 1 (1940s, 66–75 years old), group 2 (1950s, 56–65 years old), group 3 (1960s, 46–55 years old), and group 4 (≥1970s, 25–45 years old).

If the patient got examined several times a day, we always used the same scheme: we took the first measurement for analysis. Only if this measurement did not meet the quality specifications would we take the following one.

To calculate the reference bodies, all examinations were opened and we manually measured the lens thickness in 0° and 90° section.

**Figure 1:**
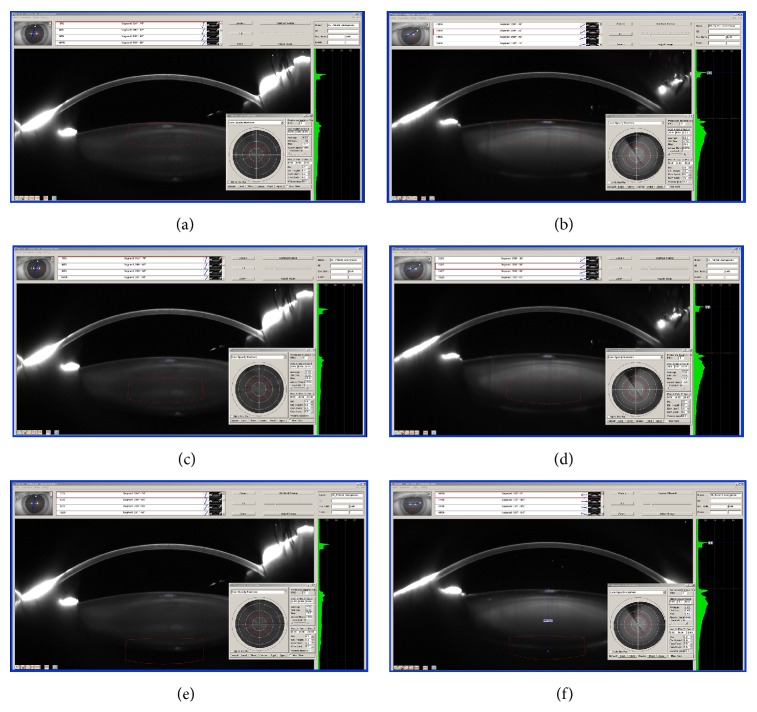
Patient sample of group 3. Three-dimensional lens densitometry before/after vitreoretinal surgery with preliminary silicone oil fill, measurements in three segments: upper line: pre-op. (left), anterior segment, post-op. (right); middle line: pre-op. (left), nucleus, post-op. (right); lower line: pre-op. (left), posterior segment, post-op. (right). Red cylinder: reference body for the three segments. Blue circle: light scattering artefact eliminated from densitometry calculations.

After this, we took the mean value of these measurements for the four cohorts. Here we recognized that there were no differences between the 1940s to 1960s age groups. So for these three groups a reference body of a diameter of 3.0 mm, a height of 1.6 mm, and a mean lens thickness of 4.23 mm was built. This reference body was used to analyse the lens densitometry for the anterior, center, and posterior segments of the lens (Figure 1). The overlap between the reference bodies constituted 0.2 mm.

For the youngest group we measured thinner lenses, so that we calculated a reference body of 3.0 mm in diameter, 1.4 mm in height, and 3.76 mm in mean lens thickness. The analysis of the lens densitometry was analogous to the other groups explained above.

### 2.3. Silicone Oil Emulsification

In the retrospective study, 80 eyes out of 101 had been filled with 5000 mPa·s (millipascal seconds) oil, 4 eyes with 4300 mPa·s oil, and 13 eyes with 2000 mPa·s oil and in 4 eyes we had no definite specification. In the prospective study we used 5000 mPa·s oil in 69 eyes, 4300 mPa·s oil in 6 eyes, and 2000 mPa·s oil in 28 eyes. From 19 eyes, silicone oils with different viscosities and peculiar appearances during the f/u were sent to a lab (alamedics GmbH & Co. KG, Dornstadt, Germany) to analyze the different severity of emulsification microscopically. The results were compared to various patient-specific factors to point out the critical ones.

Oil samples were placed immediately after oil removal on a silanized stage. A second, thinner stage was placed in a distance of 0.25 mm to create a chamber with a defined height/volume. The emulsification bubbles were counted and images were taken to allow a software to determine size and number of the bubbles per square centimeter. The results were categorized and evaluated [18].

### 2.4. Data Pool and Statistical Methods

All the analyses were performed using BiAS V10.12 [22] for Windows. *p* ≤ 0.05 was considered to indicate a statistically significant difference.

To compare the retinal redetachment rate in the prospective group with the one in the retrospective group we applied a two-tailed binomial test.

Out of 62 cases, 27 cases could meet the quality requirements of the Pentacam imaging system.

Because a standardized normal (Gaussian) distribution of parameters was not guaranteed in all groups after performing a Kolmogorov-Smirnov test, we chose a Wilcoxon matched-pairs test (with exact *p* value) for statistical evaluation of pre- and postoperative lens density. The Rosenthal effect size [23] was used regarding the clinical relevance; therefore 0.1 is seen as a small, 0.3 as a medium, and 0.5 as a large effect.

For proof of significance in lens maturation during silicone oil fill between the different age groups, a Kruskal-Wallis test with multiple Conover-Iman comparisons (Bonferroni-Holm-corrected) was performed. Additionally, for better illustration, we performed linear regression after Pearson to show the relation between patients' age and the influence of silicone oil on lens density increase.

To evaluate the influence of lens status and the interferences with the silicone oil for the prospective group, the Fisher-Freeman-Halton Exact Test for contingency tables with Valz and Thompson's algorithm was performed.

## 3. Results

### 3.1. Lens Status and Retinal Reattachment Success Rate

From 204 eyes, 101 were followed up retrospectively with 65 eyes staying stable after the oil removal with a redetachment rate of 35,64%. After having designed [16, 17] a standard operation protocol (SOP) and an evaluation protocol (EVALP), 103 eyes were followed up prospectively with 96 eyes staying permanently attached with a significantly reduced redetachment rate of 6,8% (*p* = 0.002).

The retinal reattachment rate was highest in the prospective pseudophakic group with a *p* value of 0.039.

### 3.2. Effect of Silicone Oil on Lens Maturation

As mentioned above, by calculating the reference bodies for the lens densitometry, we could detect that there was no difference in the mean value of lens thickness between the 1940s to 1960s age groups. Only for the youngest group (≥1970) we measured thinner lenses, so that we adapted the reference body. The patients' demographic data and the baseline characteristics of these four age groups, which could be finally analyzed by Scheimpflug, are listed in Table 1.

After testing the statistical significance of the lens densitometry values before and after silicone oil fill for the four age groups, all except the youngest group showed significant effects of lens maturation (*p* ≤ 0.05) with the reference body aligned through the whole lens (Table 2). We saw that the “middle aged” groups (group 2 and group 3) had the strongest maturation.

Group 3 had a significant lens maturation in all parts of the lens during the silicone oil fill. Group 2 had a highly significant maturation in the anterior and center part of the lens (*p* = 0.004). Only the posterior part was not significantly affected in group 2 (*p* = 0.375). The oldest group (group 1) reacted less compared to the “middle aged” groups. Regarding the different segments of the lens, the strongest effect was seen in the anterior part of the lens (*p* = 0.094). A potential explanation for this is that older patients (group 1) already had a more pronounced cataract before the silicone oil fill as shown in Table 2.

In contrast to the three older age groups, the youngest group is the only one who had no significance either for the whole reference body (*p* = 0.313) or for the anterior (*p* = 0.500), center (*p* = 0.438), or posterior (*p* = 0.250) reference body. The strongest effect (not significant) was seen in the posterior part for the young patients.

To confirm our finding that the “middle aged” groups had the strongest maturation effect because of silicone oil, we used the Kruskal-Wallis test [24] together with the Conover-Iman test of multiple comparisons (Bonferroni-Holm-corrected).

Considering the Kruskal-Wallis test first, there was a statistically significant effect between the different age groups for the whole (*p* = 0.017), anterior (*p* = 0.015), and center part (*p* = 0.009) of the lens.

Only for the posterior part, no significance (*p* = 0.080) could be found between the four cohorts.

Regarding the Conover-Iman test of multiple comparisons, for the whole reference body as well as for the center part, we could find a statistically significant difference in maturation between group 2 versus group 3 and group 2 versus group 4 but not between group 1 and group 4. That confirms our theory that the biggest maturation can be seen in the “middle aged” groups. The increase of lens density in the anterior part of the lens was significant in groups 2 (*p* = 0.012) and 3 (*p* = 0.050) and also in the oldest group 1 (*p* = 0.012) versus the youngest group 4.

### 3.3. Lens Densitometry in Different Lens Zones

**Figure 2:**
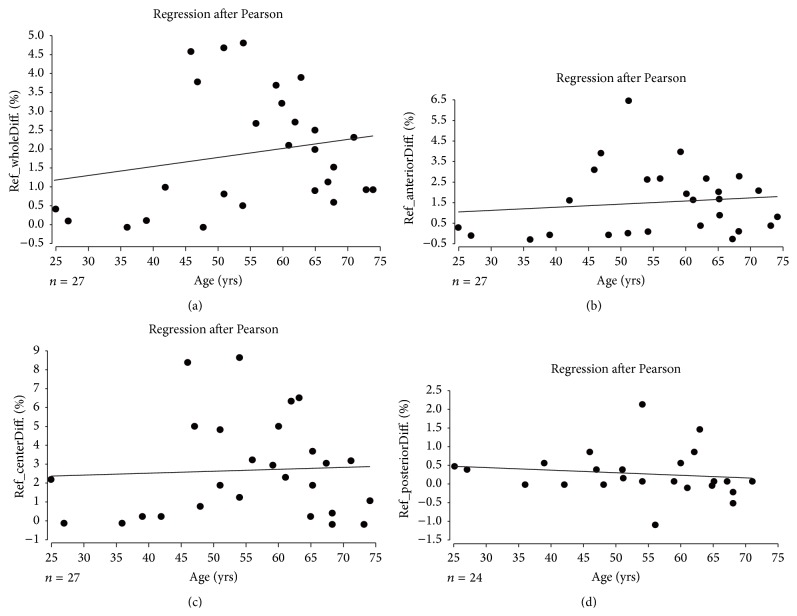
(a)–(d) Lens densitometry change in % for the reference body aligned through the whole lens (Ref_wholeDiff.) (a), for the anterior reference body (Ref_anteriorDiff.) (b), for the central reference body (Ref_centerDiff.) (c), and for the posterior reference body (Ref_posteriorDiff.) (d) with a diameter zone of 3 mm, all plotted against the age. For the posterior reference body three patients could not be adjusted.

For better illustration of the results in the different age groups, the specific changes in the different layers are plotted against the age in Figures 2(a)–2(d).

You can see that the age distribution is not a linear function, since the transparency loss increases from group 4 (youngest) to groups 3 and 2 but slows down in group 1 (oldest) as approved by the Kruskal-Wallis test above.

### 3.4. Lab Analysis of Silicone Oils

In general, the 5000 and 4300 mPa·s silicone oils proved to be more stable than the 2000 mPa·s silicone oils without showing any significance onto the outcome of retinal reattachments, visual acuity, or visual function [18].

In the lab we analyzed ten 4300 mPa·s oils, seven 5000 mPa·s oils, and two 2000 mPa·s oils after silicone oil removal. We could not find a significant difference in the severity of emulsification droplets (*p* = 0.677).

**Figure 3:**
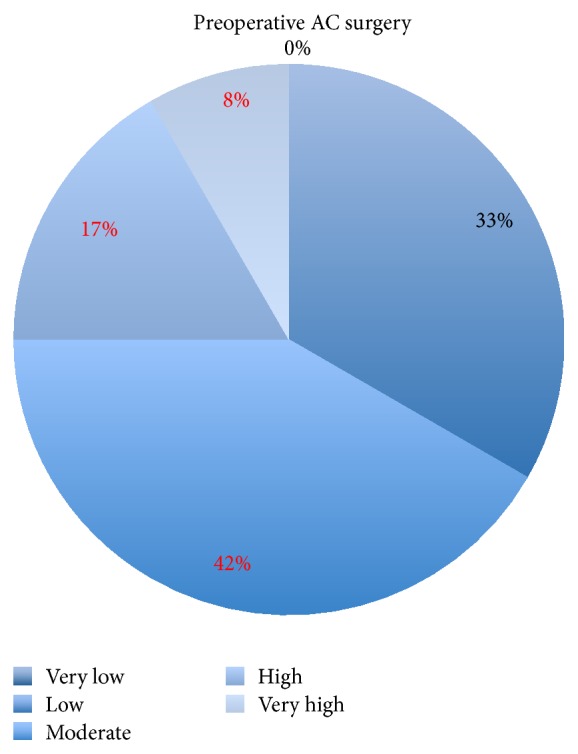
Silicone oil emulsification droplets counted 4 months after oil injection into eyes preoperated on in the anterior chamber (AC) before retina service. Highlighted is moderate (42%) plus high (17%) plus very high (8%) emulsification rate = 67%.

**Figure 4:**
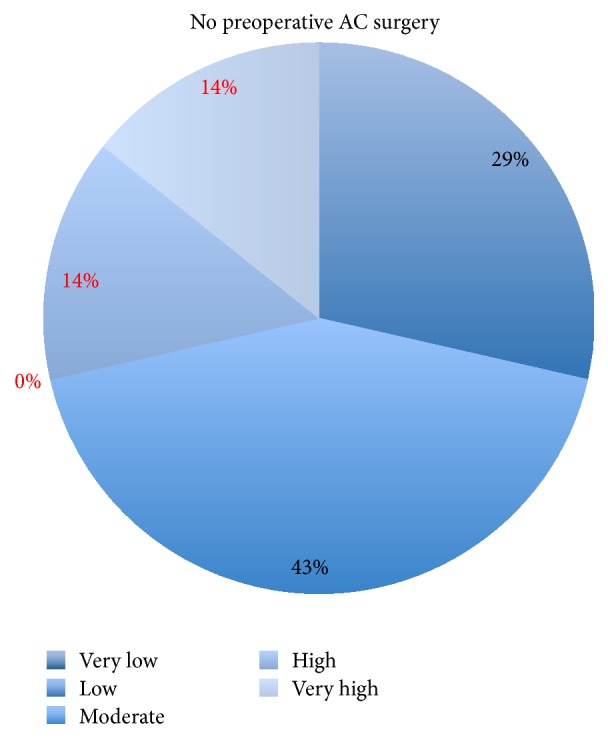
Silicone oil emulsification droplets counted 4 months after oil injection into eyes not preoperated on in the AC before retina service. Highlighted is moderate (0%) plus high (14%) plus very high (14%) emulsification rate = 28%.

Seven of 19 eyes, where the silicone oil could be lab analyzed after removal, had no preceding surgery in the anterior chamber. In 12 eyes surgery in the anterior chamber before detachment, the surgery seemed to increase the degree of all emulsification with a distinct, but not significant, trend (*p* = 0.187). For better illustration, we highlighted the percentage of moderate, high, and very high emulsification in Figures 3 and 4.

## 4. Discussion

Needless to say that, among several parameters contributing to a better viewing into the eye and its pathology, first of all, the optics attached to a high quality operating microscope should be as perfect as possible to guarantee the best viewing. This is achieved by using modern wide-field observation systems (like the Super-View System, the Resight, the BIOM, etc.) and high resolution contact lenses for the viewing through contact lenses; the transparency of media might be an even more critical issue.

Furthermore, the distribution and extent of the pathology, for example, an AHFP in diabetics, might decide on the need to sacrifice the lens in an early stage to guarantee no restrictions for membrane peeling and removal in the outermost periphery of the vitreous cavity.

As shown by us in 1994 [25], we should not be surprised about significant changes in all layers of the natural lens in human eyes once silicone oil was applied, even if only for two, four, or six months. In that study, the posterior capsule and cortex of the lenses stayed clear for quite a long time. We had expected the typically described nuclear changes but also found significant changes in the anterior capsule and the subcapsular lens epithelium. This had impeded the performance of the anterior capsular rhexis which could only be compensated by modifications of the rhexis performance strategy and technology with even the development of special electromechanical devices.

As shown in the current study, lenses responded to surgery with silicone oil in all layers, in the “middle aged” groups more than in the younger group or in the age group of 70 years and older.

It is also obvious that the changes affected the nucleus and the anterior cortex more intensively and rapidly than the posterior segment of the lens.

This is different from modern research analysis of the response to heavy silicone oil which in principle is characterized by more impurities, a higher likelihood to emulsify at an earlier time, and even the development of retrolental membranes because of its interferences with the metabolism of posterior capsule epithelial cells [26].

The microscopic examination of the lens capsule in eyes after the use of heavy silicone oil tamponades demonstrated the presence of macrophages adhering to the lens capsule with epithelioid cells and fibroblastic differentiation, thus adding a probable inflammatory genesis to cataract formation [27]. Our clinical Scheimpflug observations in the current study as well as our older data from microscopic examinations of anterior capsules after silicone oil versus trauma give hints that the release of cytokines and other substances triggered by the oil might pass through the zonula around the lens and affect its anterior surface.

Data from the microscopic paper [25] showed that when the anterior zone of the lenses got thickened, this was caused mainly by multiplication of the epithelial layer. This happened after trauma (no silicone oil) and after temporary silicone oil fill, which allows one to speculate that potentially inflammatory processes might play a role.

And indeed in literature, comparable changes [28–30] are said to be the result of a variety of disturbances such as trauma, chronic inflammation (e.g., iritis), and metabolic impairment. In our cases of silicone-filled vitreous cavities, such disturbances might have been induced by the apposition of the silicone oil to the posterior lens surface, the zonular fibers, and the iris. Besides the silicone oil, also PVR processes and/or side effects of the surgical procedure are potential cofactors.

Since modern silicone oils can lose stability through many factors, for example, the use of perfluorocarbons with incomplete removal from the eye, high energy laser coagulation, high concentrations of inflammatory cells, and less stability after anterior segment surgery, the question of whether lens surgery and vitrectomy with silicone oil outside of the macula pucker and macula hole surgery should better be separated from each other and not performed as a combined procedure in complex pathologies like P(D)VR might be raised.

Once we decide to go for a silicone oil tamponade we should be aware of the principle need for as complete as possible removal of vitreous and oil fill to reduce the risk of emulsification.

It is well known that the raw sources of silicone oil did change in the last decade which challenged all silicone oil providers to work hard on the chemical treatment to guarantee a stable quality for the use in human eyes. Further, the shift of the 19/20-gauge standard for vitrectomy instrumentations down to 23/25- or even 27-gauge standard ends up with an optional more “gentle” surgical approach which often is associated with a trend to a less complete removal of vitreous. Proliferative vitreoretinopathy (PVR) activity includes inflammatory cell activity which might even count more for the emulsification risk if more vitreous in such a PVR environment is left and brought into temporary contact with the silicone oil. This change of standard might have an impact on the tolerance for vitreous substitutes with subsequent increase of emulsification risks. Together, the increased rate of emulsification plus the higher concentrated accumulation of inflammatory cells and the reaction to this might lead to a poor functional prognosis. Costagliola et al. [31, 32] suggest that the relatively low concentration of surface-active agents only partially accounts for systematic production of emulsions; gravitational instability, originating from the interface by tangential disturbances, is of way more importance for the formation of emulsions. This makes sense from our point of view, especially when we take into consideration our hypothesis of a moderate increase of residual vitreous concentration with more inflammatory cells and more interface for the tangential disturbances as mentioned above and our observation of more air bubbles acccompanying the different oils when injected through the smaller gauge needles.

As a matter of fact, new silicone oil manufacturing issues had forced us during our retrospective-to-prospective study between 2010 and 2015 to give up on a two-port “passive” oil removal 4 months after instillation because of an increased rate of emulsification and phenomena like residual oil bubbles sticking to the retina or to the lens surface (“sticky oil”) [33]. However, once we had washed out the eye routinely via a 3-port revision surgery, the final outcome was adequate.

## 5. Conclusions

Surgery for retinal reattachment seems to benefit from preoperative cataract removal. Interestingly, we found specific lens changes not only in the nucleus and the layers posterior but also in the layers anterior to it. This corresponds to the histology of the lens epithelium published before and affects the anterior capsulorhexis maneuver.

If side effects of the preliminary silicone oil fill do affect the lens surface opposite to the silicone oil contact plane (anterior lens capsule) and modern silicone oils, no matter what type they are, developing instability earlier, we might be a bit more critical in combining anterior and posterior segment surgery in very complex pathologies and also restrict the oil fill period to the maximum time necessary to slow down active inflammatory processes. At the end of the day, an increased rate of emulsification plus the higher concentrated accumulation of inflammatory cells and the reaction to that might include a risk of poor functional prognosis.

## Figures and Tables

**Table 1:** Patient's demographic and baseline characteristics for the subgroup on which Scheimpflug photography was performed and could be analyzed after quality check.

	Group 1	Group 2	Group 3	Group 4
Number of eyes/patients	6	9	7	5
Age group	1940–49	1950–59	1960–69	≥1970
Age (yrs), mean ± SD	69.5 ± 2.93	62 ± 3.11	51 ± 3.24	36 ± 7.46
Gender, number (%)				
Male	3 (50.0)	3 (33.3)	5 (71.4)	1 (20.0)
Female	3 (50.0)	6 (66.7)	2 (28.6)	4 (80.0)
Eye, number (%)				
Right	6 (100.0)	4 (44.4)	6 (85.7)	4 (80.0)
Left	0 (0.0)	5 (55.6)	1 (14.3)	1 (20.0)
Axial length (%)				
Emmetropic	3 (50.0)	3 (33.3)	1 (14.3)	2 (40.0)
Myopic	3 (50.0)	6 (66.7)	6 (85.7)	3 (60.0)
Hyperopic	0 (0.0)	0 (0.0)	0 (0.0)	0 (0.0)
BCVA preop. (decimal)				
Mean ± SD	0.35 ± 0.33	0.1 ± 0.23	0.43 ± 0.39	0.04 ± 0.12
Range	0.0010–0.8	0.0010–0.6	0.0286–0.7	0.0010–0.3
BCVA postop. (decimal)				
Mean ± SD	0.25 ± 0.09	0.2 ± 0.11	0.07 ± 0.14	0.05 ± 0.16
Range	0.2–0.4	0.0010–0.25	0.0010–0.32	0.0010–0.4
Cataract surgery (%)				
Revision surgery	6 (100.0)	7 (77.8)	3 (42.9)	1 (20.0)
In the course	0 (0.0)	2 (22.2)	4 (57.1)	4 (80.0)
PVR stage (%)				
AB/A	1 (16.7)	3 (33.3)	4 (57.1)	0 (0.0)
C1/B	3 (50.0)	0 (0.0)	2 (28.6)	0 (0.0)
C2/C1	1 (16.7)	4 (44.4)	0 (0.0)	2 (40.0)
C3/C2	1 (16.7)	0 (0.0)	0 (0.0)	3 (60.0)
D1/C3	0	0 (0.0)	0 (0.0)	0 (0.0)
D2, D3/D1	0	2 (22.2)	1 (14.3)	0 (0.0)
Preop. fovea situation (%)				
Attached	3 (50.0)	3 (33.3)	2 (28.6)	2 (40.0)
Washed up	2 (33.3)	0 (0.0)	3 (42.9)	0 (0.0)
Detached	1 (16.7)	6 (66.7)	2 (28.6)	3 (60.0)

**Table 2:** Change in lens densitometry.

	Group 1	Group 2	Group 3	Group 4
Reference body whole lens (%)				
Preoperative mean	9.5	8.7	8.1	7.8
Preoperative range	9.1–10.3	8.2–9.5	7.7–8.7	7.5–8.1
Mean 4 months after silicone oil fill	10.85	11.4	11.7	8.0
Range 4 months after silicone oil fill	10.2–11.7	9.4–13.4	8.2–13.4	7.6–9.0
*p* value	0.031	0.004	0.031	0.313
Effect size	0.637	0.628	0.587	0.384

Reference body anterior (%)				
Preoperative mean	10.6	9.2	8.5	7.8
Preoperative range	10.0–12.1	8.8–10.2	7.9–9.6	7.5–8.4
Mean 4 months after silicone oil fill	11.7	10.2–12	11.4	8.0
Range 4 months after silicone oil fill	10.9–13.6	9.4–13.3	8.1–16.5	7.5–9.9
*p* value	0.094	0.004	0.031	0.500
Effect size	0.514	0.628	0.636	0.436

Reference body center (%)				
Preoperative mean	8.6	8.1	7.3	7.2
Preoperative range	8.0–10.5	7.7–10.7	7.2–7.8	7.1–7.7
Mean 4 months after silicone oil fill	9.8	11.0	11.7	7.5
Range 4 months after silicone oil fill	7.8–11.7	8.1–17.0	7.9–16.3	7.1–9.3
*p* value	0.156	0.004	0.016	0.438
Effect size	0.455	0.628	0.633	0.343

Reference body posterior (%)				
Preoperative mean	7.4	7.4	7.1	7.4
Preoperative range	7.2–7.6	7.2–8.3	7.1–7.6	7.2–7.5
Mean 4 months after silicone oil fill	7.2	7.4	7.7	7.6
Range 4 months after silicone oil fill	7.1–7.6	7.2–9.7	7.1–9.3	7.5–8.0
*p* value	0.625	0.375	0.031	0.250
Effect size	0.258	0.294	0.636	0.655

Statistical analysis: Wilcoxon matched-pairs test with exact *p* value. Effect size: *r* = *Z*/sqr(2*∗n*′): Rosenthal: 0.1, “small effect,” 0.3, “medium effect,” and 0.5, “large effect.”

## Abbreviations

AC: Anterior chamber
AHFG: Anterior hyaloid fibrovascular proliferation
EVALP: Evaluation protocol
f/u: Follow-up
mPa·s: Millipascal seconds
P(D)VR: Proliferative (diabetic) vitreoretinopathy
SLP: Scheimpflug lens photography
SOT: Silicone oil tamponade
SOP: Standard operation procedure.

## References

[1] Burton T. C. (1982). Recovery of visual acuity after retinal detachment involving the macula. *Transactions of the American Ophthalmological Society*.

[2] Heimann H., Hellmich M., Bornfeld N., Bartz-Schmidt K. U., Hilgers R. D., Foerster M. H. (2001). Scleral buckling versus primary vitrectomy in rhegmatogenous retinal detachment (SPR Study): design issues and implications. SPR Study report no. 1. *Graefe's Archive for Clinical and Experimental Ophthalmology*.

[3] Heimann H., Zou X., Jandeck C. (2006). Primary vitrectomy for rhegmatogenous retinal detachment: an analysis of 512 cases. *Graefe's Archive for Clinical and Experimental Ophthalmology*.

[4] Boscia F., Furino C., Recchimurzo N., Besozzi G., Sborgia G., Sborgia C. (2008). Oxane HD vs silicone oil and scleral buckle in retinal detachment with proliferative vitreoretinopathy and inferior retinal breaks. *Graefe's Archive for Clinical and Experimental Ophthalmology*.

[5] Mougharbel M., Koch F. H. J., Böker T., Spitznas M. (1994). No cataract two years after pneumatic retinopexy. *Ophthalmology*.

[6] Koch F., Kloß K. M., Hockwin O., Spitznas M. (1991). Changes in lens transparency following vitrectomy with intraocular tamponade: densitometric image analysis of Scheimpflug photographs 6 months postoperatively. *Klinische Monatsblätter für Augenheilkunde*.

[7] Johansson K., Malmsjö M., Ghosh F. (2006). Tailored vitrectomy and laser photocoagulation without scleral buckling for all primary rhegmatogenous retinal detachments. *British Journal of Ophthalmology*.

[8] Oyagi T., Emi K. (2004). Vitrectomy without scleral buckling for proliferative vitreoretinopathy. *Retina*.

[9] Siqueira R. C., Gomes C. V. M. C., Dalloul C., Jorge R. (2007). Vitrectomy with and without scleral buckling for retinal detachment. *Arquivos Brasileiros de Oftalmologia*.

[10] Pastor J. C. (1998). Proliferative vitreoretinopathy: an overview. *Survey of Ophthalmology*.

[11] Azen S. P., Scott I. U., Flynn H. W. (1998). Silicone oil in the repair of complex retinal detachments: a prospective observational multicenter study. *Ophthalmology*.

[12] Nadal J., Verdaguer P., Canut M. I. (2012). Treatment of retinal detachment secondary to macular hole in high myopia: vitrectomy with dissection of the inner limiting membrane to the edge of the staphyloma and long-term tamponade. *Retina*.

[13] Ortisi E., Avitabile T., Bonfiglio V. (2012). Surgical management of retinal detachment because of macular hole in highly myopic eyes. *Retina*.

[14] Wei Y., Li Y., Chen F. (2014). Vitrectomy treatment of retinal detachments related to choroidal coloboma involving the disk. *Retina*.

[15] Kapur R., Birnbaum A. D., Goldstein D. A. (2010). Treating uveitis-associated hypotony with pars plana vitrectomy and silicone oil injection. *Retina*.

[16] Deuchler S., Krüger H., Koss M., Singh P., Koch F. (2011). Retinal re-detachment after silicone oil removal: destiny or beat the enemy?. *Investigative Ophthalmology & Visual Science*.

[17] Deuchler S., Krüger H., Koss M., Singh P., Koch F. Retina re-detachment after silicone oil removal: destiny or beat the enemy?.

[18] Deuchler S., Singh P., Müller M. Peri- and intraoperative factors affecting the emulsification of si-licone oil used for retinal reattachment in complicated retinal detachments.

[19] Kim J.-S., Chung S.-H., Joo C.-K. (2009). Clinical application of a Scheimpflug system for lens density measurements in phacoemulsification. *Journal of Cataract and Refractive Surgery*.

[20] Kirkwood B. J., Hendicott P. L., Read S. A., Pesudovs K. (2009). Repeatability and validity of lens densitometry measured with Scheimpflug imaging. *Journal of Cataract & Refractive Surgery*.

[21] Mayer W. J., Klaproth O. K., Hengerer F. H., Kohnen T. (2014). Impact of crystalline lens opacification on effective phacoemulsification time in femtosecond laser-assisted cataract surgery. *American Journal of Ophthalmology*.

[22] Ackermann H. (1991). A program package for biometrical analysis of samples. *Computational Statistics & Data Analysis*.

[23] Rosenthal R. (1991). *Meta-Analytic Procedures for Social Research*.

[24] Kruskal W. H., Wallis W. A. (1953). Use of ranks in one-criterion variance analysis. *Journal of the American Statistical Association*.

[25] Koch F. H. J., Cusumano A., Seifert P., Mougharbel M., Augustin A. J. (1995). Ultrastructure of the anterior lens capsule after vitrectomy with silicone oil injection. Correlation of clinical and morphological features. *Documenta Ophthalmologica*.

[26] Hiscott P., Magee R. M., Colthurst M., Lois N., Wong D. (2001). Clinicopathological correlation of epiretinal membranes and posterior lens opacification following perfluorohexyloctane tamponade. *British Journal of Ophthalmology*.

[27] Leaver P. K., Grey R. H., Garner A. (1979). Complications following silicone oil injection. *Modern Problems in Ophthalmology*.

[28] Yanoff M., Fine B. S. (1975). Lens. *Ocular Pathology*.

[29] Spencer W. H. (1985). Chapter 5: lens. *Ophthalmic Pathology*.

[30] Pau H., Novotny G. E. K., Arnold G. (1985). Ultrastructural investigation of extracellular structures in subcapsular white corrugated cataract (anterior capsular cataract). *Graefe's Archive for Clinical and Experimental Ophthalmology*.

[31] Costagliola C., Semeraro F., Dell'Omo R. (2014). Some physicochemical remarks on spontaneous emulsification of vitreal tamponades. *BioMed Research International*.

[32] Barca F., Caporossi T., Rizzo S. (2014). Silicone oil: different physical proprieties and clinical applications. *BioMed Research International*.

[33] Dresp J. H., Menz D.-H. (2007). The phenomenon of ‘sticky’ silicone oil. *Graefe's Archive for Clinical and Experimental Ophthalmology*.

